# Supplementary material for: Cultural Adaptation and Psychometric Properties of the Trust Me Scale—Italian Version: A Validation Study
Source: Healthcare (Basel). 2023 Apr 11;11(8):1086. doi: 10.3390/healthcare11081086 (PMC10137678; doi:10.3390/healthcare11081086)
Supplement: Supplementary file 1 [file healthcare-11-01086-s001.zip › healthcare-2277675-supplementary.pdf]

Suplementary Materials

Cultural Adaptation and Psychometric Properties of the Trust Me Scale—Italian Version: A Validation Study

Dhurata Ivziku [1], Rosario Caruso [2,3] Marzia Lommi*[4,] Gianluca Conte [2], Arianna Magon [2], Alessandro Stievano[5] , Gennaro Rocco[5] , Ippolito Notarnicola[5] , Maddalena De Maria[6], Raffaella Gualandi[1], Daniela Tartaglini[1] and Anna De Benedictis[1,7]

Table S1. Trust Me Scale Italian-Nurse Version

	Le seguenti affermazioni valutano la **fiducia tra gruppo e coordinatore**. Indichi quale risposta rappresenta al meglio la sua opinione.	Fortemente in disaccordo	In disaccordo	Neutrale	D' accordo	Fortemente d' accordo
1	I bisogni e i desideri del gruppo sono molto importanti per il coordinatore.	1	2	3	4	5
2	Posso contare sul mio coordinatore per aiutarmi se sono in difficoltà con il lavoro.	1	2	3	4	5
3	Il coordinatore non farebbe consapevolmente nulla per danneggiare l'azienda.	1	2	3	4	5
4	Il mio coordinatore è aperto e sincero con me.	1	2	3	4	5
6	Il coordinatore mantiene gli impegni presi.	1	2	3	4	5
7	Il coordinatore sta davvero attento a ciò che è importante per il gruppo.	1	2	3	4	5
8	Il coordinatore è consapevole del lavoro che deve essere svolto.	1	2	3	4	5
9	Il coordinatore è noto per avere successo nelle cose che tenta di realizzare.	1	2	3	4	5
10	Se commetto un errore, il mio coordinatore è disposto a "perdonare e dimenticare".	1	2	3	4	5
12	Il coordinatore intraprende azioni coerenti con gli impegni assunti.	1	2	3	4	5
14	C'è molto calore nelle relazioni tra i coordinatori e i lavoratori di questa organizzazione.	1	2	3	4	5
15	Il coordinatore farebbe sacrifici personali per il nostro gruppo.	1	2	3	4	5
16	Il coordinatore esprime i suoi veri sentimenti su questioni importanti.	1	2	3	4	5

Table S2. Trust Me Scale Italian-Nurse manager Version

	Le seguenti affermazione valutano la **fiducia tra gruppo e leader**. Indichi quale risposta rappresenta al meglio la sua opinione.	Fortemente in [illegible]	In disaccordo	Neutrale	D’ accordo	Fortemente d’ accordo
1	I bisogni e i desideri del gruppo sono molto importanti per il coordinatore.	1	2	3	4	5
2	Posso contare sul mio gruppo per aiutarmi se sono in difficoltà con il lavoro.	1	2	3	4	5
3	Il gruppo non farebbe consapevolmente nulla per danneggiare l'azienda.	1	2	3	4	5
4	I mio gruppo è aperto e sincero con me.	1	2	3	4	5
6	Il gruppo mantiene gli impegni presi.	1	2	3	4	5
7	Il gruppo sta davvero attento a ciò che è importante per il coordinatore.	1	2	3	4	5
8	Il gruppo è consapevole del lavoro che deve essere svolto.	1	2	3	4	5
9	Il gruppo è noto per avere successo nelle cose che tenta di realizzare.	1	2	3	4	5
10	Se commetto un errore, il mio gruppo è disposto a "perdonare e dimenticare".	1	2	3	4	5
12	Il gruppo intraprende azioni coerenti con gli impegni assunti.	1	2	3	4	5
14	C'è molto calore nelle relazioni tra i coordinatori e i lavoratori di questa azienda.	1	2	3	4	5
15	I dipendenti farebbero sacrifici personali per il nostro gruppo.	1	2	3	4	5
16	Il gruppo esprime i suoi veri sentimenti su questioni importanti.	1	2	3	4	5
